# Prognostic Analysis of Different Metastatic Patterns in Invasive Intraductal Papillary Mucinous Neoplasm: A Surveillance, Epidemiology, and End Results Database Analysis

**DOI:** 10.1155/2021/4350417

**Published:** 2021-12-23

**Authors:** Chen Zhou, Zhiqiang Liu, Yingke Zhou, Dianyun Ren, Kun Liu, Gengdu Qin, Huan Zhang, Xueyi Liang, Shanmiao Gou, Heshui Wu

**Affiliations:** Department of Pancreatic Surgery, Union Hospital, Tongji Medical College, Huazhong University of Science and Technology, Wuhan 430022, China

## Abstract

**Objective:**

To evaluate the impacts of different metastatic patterns on the prognosis of patients with invasive intraductal papillary mucinous neoplasm (IPMN).

**Materials and Methods:**

All patients who were diagnosed with invasive IPMN in the Surveillance, Epidemiology, and End Results SEER database (2010–2015) were included in this study. They were grouped according to different metastatic patterns. Kaplan–Meier analysis and log-rank test were used for the comparison of their survival rates. The hazard ratio (HR) with 95% confidence interval (CI) was analyzed using the Cox proportional-hazards model.

**Results:**

A total of 2264 cases were included in this study. The most common metastatic site was the liver. The patients with the nonorgan metastasis demonstrated the best survival outcomes, while those with multiple metastases showed the worst survival outcomes. As compared to the patients with isolated liver metastasis, those with isolated lung and other organ metastases showed better overall survival rates and tumor-specific survival rates. The patients with liver, lung, multiple, and other organ metastases or of age >60 years were the independent predictors of poor prognosis.

**Conclusions:**

The patients with isolated lung and other organ metastases demonstrated better survival outcomes as compared to those with isolated liver metastasis. The patients with nonorgan metastasis demonstrated the best survival outcomes, while those with multiple metastases showed the worst survival outcomes. Further studies are needed to determine a highly selected subset of patients, who might benefit from surgery or chemotherapy.

## 1. Introduction

Ohashi et al. first described the intraductal papillary mucinous neoplasm (IPMN) about 30 years ago [1]. Due to the innovations in imaging technology and its expanded use, pancreatic cysts are easily detected and the incidences of IPMNs are also increasing [2]. Recently, IPMNs have become one of the most dramatic pancreatic tumors [3]. Currently, IPMNs represent 25% of all the cystic neoplasms of the pancreas, with an assumed incidence of 0.8 per 100,000 [4]. IPMNs are the most common of all the cystic tumors of the pancreas; branching IPMNs develop cancer in only 30% of the cases, but the main duct and mixed IPMNs have a 70% risk of becoming malignancies [5]. Invasive carcinomas, arising in or accompanying IPMN, can be of various types [6].

The primary treatment for the invasive IPMNs is surgical resection [7]. For patients with metastatic cancers, the primary treatment is antitumor therapy and palliative care. Despite the availability of various types of comprehensive therapies, the 5-year survival rates of the patients with invasive IPMNs are still poor [8, 9].

As compared to the common pancreatic ductal adenocarcinoma (PDAC), the invasive IPMNs demonstrate a more favorable prognosis [10, 11] and appear to be more indolent than the conventional PDAC [12]. For the invasive IPMNs, the American Joint Committee on Cancer (AJCC) staging classification is appropriate, where the 7th edition of staging classification is more applicable than the 8th edition [4]. However, the biological behaviors of invasive IPMNs are different from those of the PDAC [12, 13]. Previous studies have analyzed the prognostic impacts of different metastatic patterns on patients with PDAC and pancreatic neuroendocrine tumors (pNETs) [14, 15]. However, the studies focusing on the metastatic patterns of invasive IPMNs are limited. Therefore, in this study, the prognostic potential of different metastatic patterns of the patients with invasive IPMN based on the SEER database was evaluated.

## 2. Materials and Methods

### 2.1. Data

The data from the SEER database is a well-designed electronic medical record database for cancer research. Use SEER *∗* Stat software (version 8.3.4; National Cancer Institute, Bethesda, MD, USA) to obtain patient demographic data, clinical tumor characteristics, the first course of treatment, and follow-up data of life status from the SEER database. Because this study was based on a publicly available database, it was exempted from IRB approval [16].

**Figure 1:**
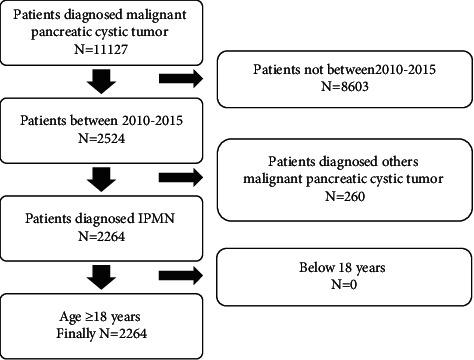
Flowchart of patients' cohort selection.

We collected patients diagnosed with invasive IPMN that were reported to the SEER database from 2010 to 2015. The case with ICD-O-3 (International Classification of Diseases for Oncology, 3rd edition) histology/behavior codes 8050, 8260, 8450, 8453, 8471, 8480, 8481, and 8503 were used to identify IPMNs. We include these pancreatic anatomical sites (C25.0, C25.1, C25.2, C25.3, C25.4, C25.7, C25.8, C25.9), and the data extraction process is shown in Figure 1.

### 2.2. Variable Definition

The variable included gender, age at diagnosis, race, marital status, tumor site, surgery, chemotherapy, radiation, grade, cancer-specific death, survival months, and vital status. The cancer-specific survival rate (CCS) is calculated based on the date of death associated with IPMN. Deaths attributed to other causes are considered to be censored observations; we divide the cases into the following groups according to the location of tumor metastasis. Since there is only 1 patient with isolated brain metastasis, this group was excluded, and we divided these cases into 6 groups.

1. Nonorgan metastasis
2. Isolated liver metastasis
3. Isolated lung metastasis
4. Isolated bone metastasis
5. Multiple (at least two organs have been metastases in the liver, lung, bone, and brain)
6. Other organ metastasis (AJCC 8th edition staging classification in stage M1, but not metastases in the above sites)

### 2.3. Statistical Analysis

Continuous variables were compared using Student's *t*-test, whereas categorical variables were compared using the chi-square test. We used Kaplan–Meier analysis and log-rank test to compare the survival rates. The Cox proportional model was employed to calculate the hazard ratio (HR) with 95% CI. *P* < 0.05 was considered statistically significant. The statistical analysis was performed using the software SPSS 24.0 (IBM, NY, United States). Proportional-hazards assumption was performed using the software STATA 16.0.

## 3. Results

### 3.1. Patients' Characteristics

In this study, a total of 2264 patients were included, whose baseline characteristics are given in Table 1. Among them, 1228 (54.25%) patients had no organ metastasis, while among those with organ metastasis, the liver was the most common isolated metastatic organ (435 (19.21%)) (Table 1), followed by other organs metastases 221 (9.76%), isolated lung metastasis 185 (8.17%), multiple organ metastases 175 (7.73%), and isolated bone metastasis 19 (0.84%). Only 1 (0.04%) patient was diagnosed with isolated brain metastasis.

### 3.2. Survival Outcomes

**Figure 2:**
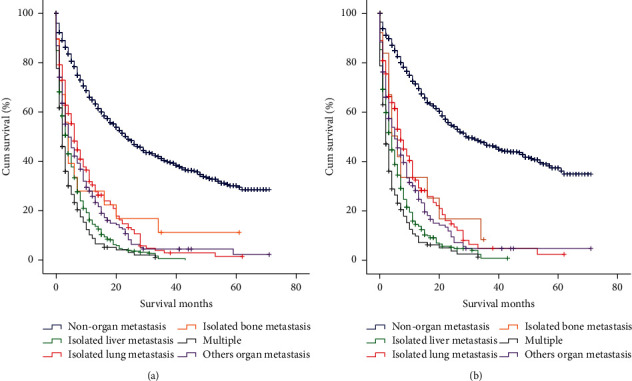
Kaplan–Meier curves and log-rank test for overall survival (a) and cancer-specific survival (b) according to different metastasis. For OS (a): nonorgan metastasis vs. other groups, *P* < 0.001; isolated liver vs. isolated lung metastasis, *P* < 0.001; isolated liver vs. isolated bone metastasis, *P*=0.048; isolated liver vs. multiple metastases, *P*=0.007; isolated liver vs. other organ metastasis, *P*=0.001; isolated lung vs. isolated bone metastasis, *P*=0.739; isolated lung vs. multiple metastases, *P* < 0.001; isolated lung vs. other organ metastasis, *P*=0.151; isolated bone vs. multiple metastases, *P*=0.019; isolated bone vs. other organ metastasis, *P*=0.486; multiple vs. other organ metastasis, *P* < 0.001. For CSS (b): nonorgan metastasis vs. other groups, *P* < 0.001; isolated liver vs. isolated lung metastasis, *P* < 0.001; isolated liver vs. isolated bone metastasis, *P*=0.078; isolated liver vs. multiple metastases, *P*=0.011; isolated liver vs. other organ metastasis, *P*=0.002; isolated lung vs. isolated bone metastasis, *P*=0.824; isolated lung vs. multiple metastases, *P* < 0.001; isolated lung vs. other organ metastasis, *P*=0.143; isolated bone vs. multiple metastases, *P*=0.019; isolated bone vs. other organ metastasis, *P*=0.487; multiple vs. other organ metastasis, *P* < 0.001.

The overall survival (OS) and cancer-specific survival (CSS) of the different metastatic sites are shown in Figure 2. The patients with multiple organs metastases demonstrated the worst survival outcomes, while those with nonorgan metastasis had the best survival outcomes. Moreover, as compared to the patients with isolated liver metastasis, those with isolated lung metastasis and other organ metastasis showed better OS and CSS (for OS, lung vs. liver metastasis *P* < 0.0001 and others organ vs. liver metastasis *P*=0.001 and for CSS, lung vs. liver metastasis *P* < 0.0001 and others organ vs. liver metastasis *P*=0.002).

**Figure 3:**
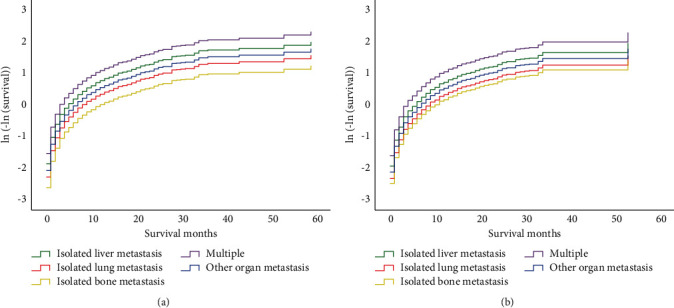
Graphically assessed proportional hazards assumption for OS and CCS based on variables of metastasis. (a) ln (-ln (survival)) for OS. (b) ln (-ln (survival)) for CCS.

The multivariate analysis revealed that the age of <60 years, yellow race, surgery, and chemotherapy were associated with better OS and CSS (Table 2). Furthermore, as compared to the patients with isolated liver metastasis, those with isolated lung metastasis and other organ metastasis showed better OS and CSS (Table 2), which were consistent with the previous results. The proportional hazards (PH) assumption test was performed using STATA v16.0; the *P* values are given in Table 2. *P* values for the PH results of the integrity of two Cox regression models were not significant, which could not be considered as a violation of the PH assumption test. Besides, the PH assumption test for OS and CCS based on the variables of metastasis (Figure 3) showed that the curve of the ln (−ln (survival probability)) tended to be parallel in the different metastatic groups. Consequently, the PH hypothesis of this variable was considered valid.

Moreover, the median survival time (MST) of the isolated lung metastasis was 6 months, followed by the other organ, isolated liver, and isolated bone metastases (4 months each). The MST of the multiple organs metastases was only 2 months.

### 3.3. Treatment Modality

**Figure 4:**
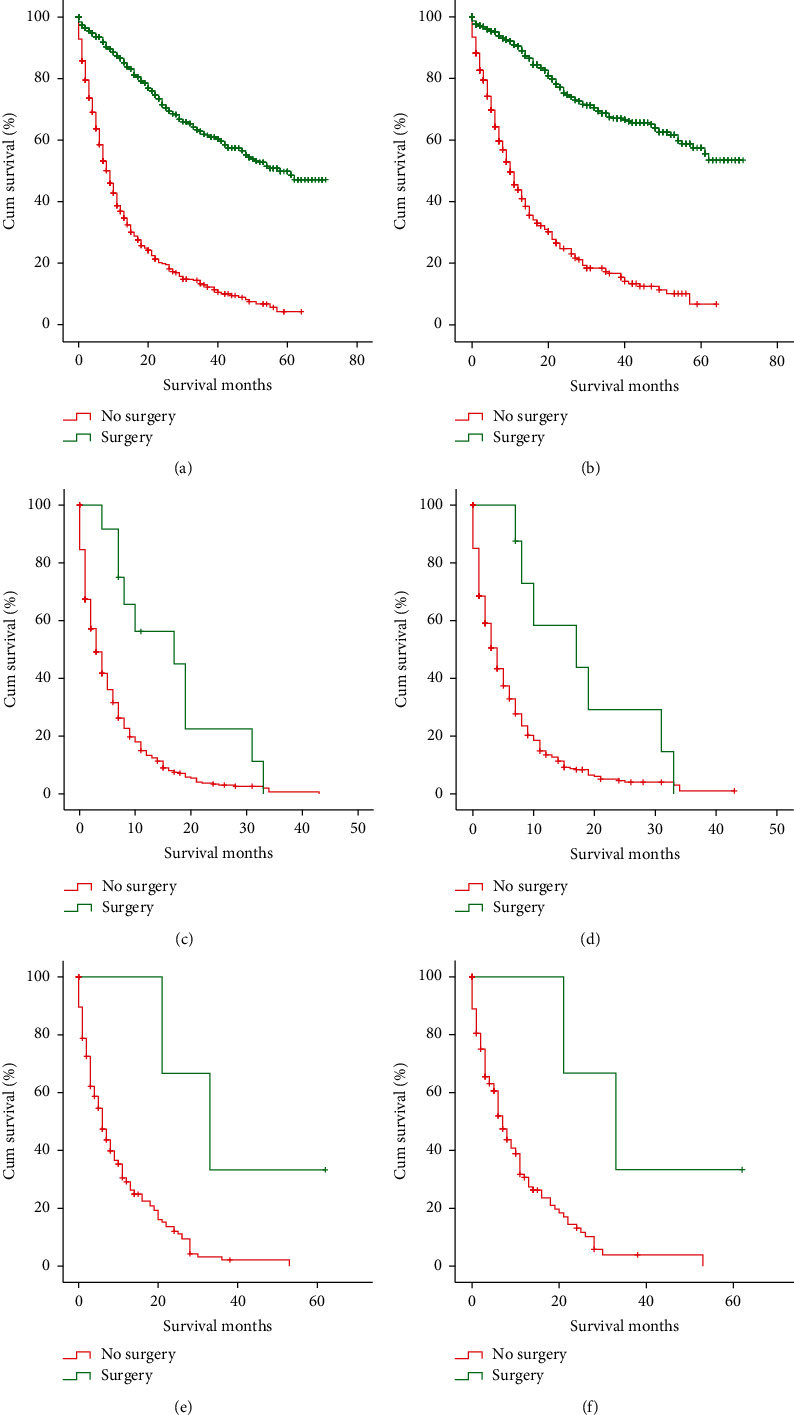
Kaplan–Meier curves and log-rank test for OS and CCS based on whether or not surgery was performed. (a) OS of nonorgan metastasis (*P* < 0.001). (b) CCS of nonorgan metastasis (*P* < 0.001). (c) OS of isolated liver metastasis (*P*=0.002). (d) CCS of isolated liver metastasis (*P*=0.011). (e) OS of isolated lung metastasis (*P*=0.012). (f) CCS of isolated lung metastasis (*P*=0.019). OS, overall survival; CCS, cancer-specific survival.

**Figure 5:**
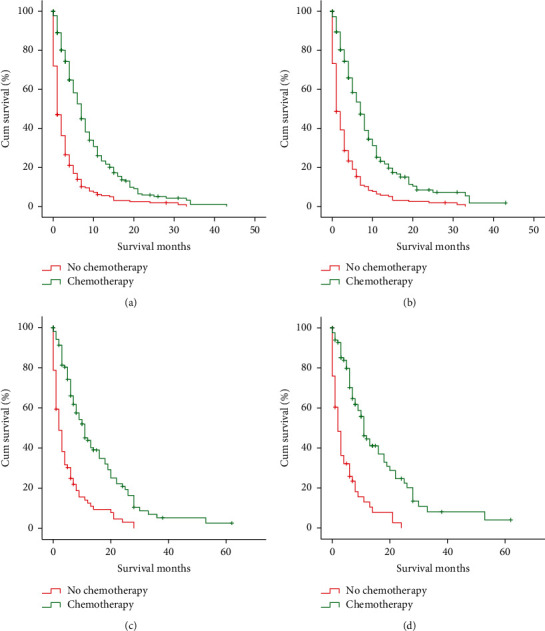
Kaplan–Meier curves and log-rank test for OS and CCS based on whether or not chemotherapy was performed. (a) OS of isolated liver metastasis (*P* < 0.0011). (b) CCS of isolated liver metastasis (*P* < 0.001). (c) OS of isolated lung metastasis (*P* < 0.001). (d) CCS of isolated lung metastasis (*P* < 0.001). OS, overall survival; CCS, cancer-specific survival.

**Figure 6:**
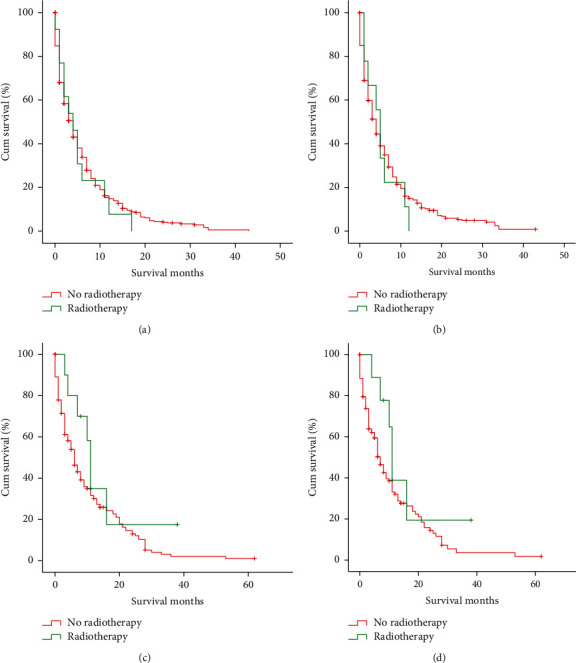
Kaplan–Meier curves and log-rank test for OS and CCS based on whether or not radiotherapy was performed. (a) OS of isolated liver metastasis (*P*=0.701). (b) CCS of isolated liver metastasis (*P*=0.731). (c) OS of isolated lung metastasis (*P*=0.158). (d) CCS of isolated lung metastasis (*P*=0.171). OS, overall survival; CCS, cancer-specific survival.

The treatment modalities are given in Table 1. A total of 728 (32.2%) patients underwent surgery, while 1536 (67.8%) patients did not undergo surgery. The surgery for the patients with distant metastasis was mainly incisional, needle, or aspiration biopsy of the primary site and/or other than primary site or palliative surgery. About half of the patients (1068, 47.2%) received chemotherapy and a few (292, 12.9%) patients received radiotherapy. The patients who underwent surgery or chemotherapy showed significant improvement in OS and CSS for those having nonorgan, isolated liver, and isolated lung metastases (Figures 4 and 5). However, the radiotherapy could not improve the 5-year OS or CSS of the patients with isolated liver and isolated lung metastases (Figure 6).

## 4. Discussion

In this study, the prognostic impacts of different metastatic patterns on the invasive IPMNs were investigated. The results showed that the patients with isolated lung metastasis or other organ metastasis demonstrated better survival outcomes than those having isolated liver metastasis, while those with multiple organ metastases showed the worst survival outcomes. These results were consistent with those in PDAC [17]. A previous study showed that adjuvant therapy for the invasive IPMNs could not improve the survival outcomes in patients with early stage metastasis [12]. However, in this study, the surgery or chemotherapy could improve the survival outcomes in metastatic invasive IPMNs (Figure 2). For the stage IV PDAC, chemotherapy was the primary choice when the performance status allowed. In clinical practice, therapy principles for the invasive IPMNs are considered as PDAC. However, the biological behaviors of invasive IPMNs are different from those of PDAC [12, 13], and fewer studies have focused on the efficacy of adjuvant therapy in stage IV invasive IPMN.

Furthermore, the prognosis of the patients with isolated lung metastasis was better than those with other organ metastasis, which might be due to the more effectiveness of chemotherapy or surgery for the isolated lung metastasis [18] or due to the fewer complications in isolated lung metastasis. Moreover, the isolated lung metastasis had better OS and CSS as compared to the isolated liver metastasis, which was consistent with the results of a previous study on PDAC [15]. This was the first study focusing on the effects of metastatic patterns on the invasive IPMNs.

The mechanisms of tumor metastasis mainly include direct invasion, lymphatic metastasis, and blood metastasis. Specifically, the metastatic process is closely related to the cross-talk between cancer and the vascular and/or lymphatic system. The normalization of tumor blood vessels can improve the infiltration of T cells, enhance the immune response and immune reaction, and halt the immune-suppressing environment to a more immune-activating phenotype and work together with cancer immunotherapy [19]. Antivascular endothelial growth factor receptor (anti-VEGFR) was used to normalize the tumor vascular system and restore its function and fostered further investigations, aiming at the formation of intratumoral immune cell phenotypes parallel to the normalization of blood vessels, as indicated by the reduction of tissue perfusion and intratumoral hypoxia [19, 20]. The anti-VEGFR polarizes the macrophages in the M1 macrophage by altering the gene expressions at the same time parallel to an increase in the adaptive immune cells' infiltration in the setting of this antiangiogenic treatment. Vascular endothelial growth factor (VEGF) and inflammatory molecules are not only the key proangiogenic elements but also act as immunomodulators. They promote the formation of blood vessels in most of the fatal malignant tumors and collaboratively create a permissive environment, resulting in the poor efficacy and survival of the patients. Cancer cells grow and progress by continuously interfering with the neighboring environment during their growth and progression. The combination of strategies, such as antiangiogenesis and immune-directed therapy, might shape the tumor ecosystem and improve the therapeutic effect [19].

In this study, there are some limitations too. First, the results should be interpreted with caution, given the inherent bias of a retrospective study. Second, the missing information on related comorbidities as well as the absence of therapy might have affected the results. Furthermore, the surgeries and chemotherapies were performed in the patients with longer life expectancies. However, this was the first study with the largest sample size to clarify the prognosis of patients with the metastatic invasive IPMN.

## 5. Conclusion

The patients with isolated lung and other organ metastases demonstrated better survival outcomes as compared to those with isolated liver metastasis, while the patients with nonorgan metastasis demonstrated the best survival outcomes and those with multiple metastases showed the worst survival outcomes. Further studies are needed to determine a highly selected subset of patients who might benefit from surgery or chemotherapy.

## Figures and Tables

**Table 1:** Baseline and clinical characteristics of the study population.

	No. (%) of patients							
Characteristics	Nonorgan metastasis (*n* = 1228)	Isolated liver metastasis (*n* = 435)	Isolated lung metastasis (*n* = 185)	Isolated bone metastasis (*n* = 19)	Multiple^*∗*^ (*n* = 175)	Other organ metastasis^*∗*^ (*n* = 221)	Total (*n* = 2263)	*P* value
Gender								0.107
Male	639 (52.0)	218 (50.1)	82 (44.3)	9 (47.4)	76 (43.4)	101 (45.7)	1125 (49.7)	
Female	589 (48.0)	217 (49.9)	103 (55.7)	10 (52.6)	99 (56.6)	120 (54.3)	1138 (50.3)	

Age (y)								0.097
<60	243 (19.8)	112 (25.7)	36 (19.5)	2 (10.5)	41 (23.4)	51 (23.1)	485 (21.4)	
≥60	985 (80.2)	323 (74.3)	149 (80.5)	17 (89.5)	134 (76.6)	170 (76.9)	1778 (78.6)	

Race								0.034
White	956 (77.9)	347 (79.8)	153 (82.7)	18 (94.7)	135 (77.1)	174 (78.7)	1783 (78.7)	
Black	116 (9.4)	47 (10.8)	19 (10.3)	0 (0.0)	22 (12.6)	33 (14.9)	237 (10.5)	
Yellow	147 (12.0)	41 (9.4)	13 (7.0)	1 (5.3)	18 (10.3)	14 (6.3)	234 (10.3)	
Unknown	9 (0.7)	0 (0.0)	0 (0.0)	0 (0.0)	0 (0.0)	0 (0.0)	9 (0.4)	

Marital								0.119
Married	696 (56.7)	219 (50.3)	105 (56.8)	10 (52.6)	94 (53.7)	127 (57.5)	1251 (55.3)	
Unmarried	470 (38.3)	194 (44.6)	63 (34.1)	9 (47.4)	73 (41.7)	85 (38.5)	894 (39.5)	
Unknown	62 (5.0)	22 (5.1)	17 (9.2)	0 (0.0)	8 (4.6)	9 (4.1)	118 (5.2)	

Primary site								<0.001
Head	704 (57.3)	120 (27.6)	65 (35.1)	6 (31.6)	39 (22.3)	46 (20.8)	980 (43.3)	
Body/tail	270 (22.0)	168 (38.6)	65 (35.1)	8 (42.1)	77 (44.0)	84 (38.0)	672 (29.7)	
Others	254 (20.7)	147 (33.8)	55 (29.7)	5 (26.3)	59 (33.7)	91 (41.2)	611 (27.0)	

Surgery								<0.001
No	532 (43.3)	423 (97.2)	182 (98.4)	18 (94.7)	174 (99.4)	206 (93.2)	1535 (67.8)	
Yes	696 (56.7)	12 (2.8)	3 (1.6)	1 (5.3)	1 (0.6)	15 (6.8)	728 (32.2)	

Chemotherapy								<0.001
No	720 (58.6)	215 (49.4)	80 (43.2)	10 (52.6)	82 (46.9)	88 (39.8)	1195 (52.8)	
Yes	508 (41.4)	220 (50.6)	105 (56.8)	9 (47.4)	93 (53.1)	133 (60.2)	1068 (47.2)	

Radiotherapy								<0.001
No	1008 (82.1)	422 (97.0)	175 (94.6)	8 (42.1)	149 (85.1)	209 (94.6)	1971 (87.1)	
Yes	220 (17.9)	13 (3.0)	10 (5.4)	11 (57.9)	26 (14.9)	12 (5.4)	292 (12.9)	

Grade								<0.001
I	199 (16.2)	11 (2.5)	11 (5.9)	3 (15.8)	8 (4.6)	8 (3.6)	240 (10.6)	
II	263 (21.4)	44 (10.1)	13 (7.0)	2 (10.5)	12 (6.9)	23 (10.4)	357 (15.8)	
III	105 (8.6)	29 (6.7)	7 (3.8)	0 (0.0)	13 (7.4)	19 (8.6)	173 (7.6)	
IV	15 (1.2)	2 (0.5)	0 (0.0)	0 (0.0)	0 (0.0)	2 (0.9)	19 (0.8)	
Unknown	646 (52.6)	349 (80.2)	154 (83.2)	14 (73.7)	142 (81.1)	169 (76.5)	1474 (65.1)	

MST^*∗*^ (month)	24	4	6	4	2	4	9	

^*∗*^Multiple mean metastases in at least 2 of the four organs (liver, lung, bone, and brain).^*∗*^Other organ metastasis mean metastases organs other than the liver, lungs, bones, and brain. ^*∗*^MST mean median survival time.

**Table 2:** Multivariate analyses of overall survival and pancreatic cancer-specific survival in patients with intraductal papillary mucinous neoplasm.

Features	OS		CSS	
	HR (95%CI)	*P* value	HR (95%CI)	*P* value
Metastasis				
Isolated liver metastasis	1 (reference)		1 (reference)	
Isolated lung metastasis	0.681 (0.564–0.822)	＜0.001	0.682 (0.545–0.852)	0.001
Isolated bone metastasis	0.469 (0.280–0.786)	0.004	0.551 (0.299–1.016)	0.056
Multiple	1.325 (1.098–1.600)	0.003	1.331 (1.080–1.640)	0.007
Other organ metastasis	0.784 (0.659–0.933)	0.006	0.785 (0.646–0.953)	0.015

Gender				
Male	1 (reference)		1 (reference)	
Female	1.014 (0.914–1.124)	0.796	1.007 (0.892–1.137)	0.909

Age (y)				
＜60	1 (reference)		1 (reference)	
≥60	1.347 (1.186–1.530)	＜0.001	1.271 (1.102–1.467)	0.001

Race				
White	1 (reference)		1 (reference)	
Black	0.950 (0.806–1.120)	0.542	0.884 (0.728–1.073)	0.213
Yellow	0.766 (0.639–0.920)	0.004	0.746 (0.605–0.919)	0.006

Marital				
Married	1 (reference)		1 (reference)	
Unmarried	1.107 (0.992–1.236)	0.069	1.129 (0.991–1.285)	0.067
Unknown	1.098 (0.867–1.391)	0.439	1.082 (0.828–1.414)	0.563

Primary Site				
Head	1 (reference)		1 (reference)	
Body/tail	1.079 (0.954–1.221)	0.228	1.155 (0.997–1.339)	0.055
Others	1.086 (0.955–1.234)	0.209	1.117 (0.960–1.299)	0.153

Surgery				
No	1 (reference)		1 (reference)	
Yes	0.201 (0.171–0.238)	<0.001	0.187 (0.153–0.230)	<0.001

Chemotherapy				
No	1 (reference)		1 (reference)	
Yes	0.594 (0.530–0.666)	<0.001	0.584 (0.511–0.668)	<0.001

Radiotherapy				
No	1 (reference)		1 (reference)	
Yes	1.079 (0.916–1.270)	364	1.168 (0.962–1.419)	0.116

Grade				
I	1 (reference)		1 (reference)	
II	1.220 (0.974–1.528)	0.084	1.189 (0.909–1.555)	0.207
III	1.357 (1.045–1.762)	0.022	1.315 (0.971–1.782)	0.077
IV	1.246 (0.606–2.559)	0.549	1.059 (0.385–2.909)	0.912
Unknown	0.939 (0.771–1.145)	0.536	0.892 (0.703–1.132)	0.347

For the Cox proportional model of OS, the *P* value of test proportional hazards assumption was 0.1205 (*P* > 0.05). For the Cox proportional model of CSS, the *P* value of test proportional hazards assumption was 0.0521 (*P* > 0.05).

## Data Availability

The data analyzed during the current study are available in the Surveillance, Epidemiology, and End Results (SEER) database of the National Cancer Institute (https://seer.cancer.gov/data/).
